# A Bayesian Generalized Explanatory Item Response Model to Account for Learning During the Test

**DOI:** 10.1007/s11336-021-09786-x

**Published:** 2021-08-30

**Authors:** José H. Lozano, Javier Revuelta

**Affiliations:** Universidad Autónoma de Madrid, Madrid, Spain

**Keywords:** componential models, learning models, item response theory, Bayesian estimation

## Abstract

The present paper introduces a new explanatory item response model to account for the learning that takes place during a psychometric test due to the repeated use of the operations involved in the items. The proposed model is an extension of the operation-specific learning model (Fischer and Formann in Appl Psychol Meas 6:397–416, 1982; Scheiblechner in Z für Exp Angew Psychol 19:476–506, 1972; Spada in Spada and Kempf (eds.) Structural models of thinking and learning, Huber, Bern, Germany, pp 227–262, 1977). The paper discusses special cases of the model, which, together with the general formulation, differ in the type of response in which the model states that learning occurs: (1) correct and incorrect responses equally (non-contingent learning); (2) correct responses only (contingent learning); and (3) correct and incorrect responses to a different extent (differential contingent learning). A Bayesian framework is adopted for model estimation and evaluation. A simulation study is conducted to examine the performance of the estimation and evaluation methods in recovering the true parameters and selecting the true model. Finally, an empirical study is presented to illustrate the applicability of the model to detect learning effects using real data.

**Supplementary Information:**

The online version contains supplementary material available at 10.1007/s11336-021-09786-x.

## Introduction

Learning effects may take place in educational and psychological testing when the items share a set of solution principles that can be extrapolated from one item to another, so examinees may learn to respond more effectively during the test. There is a wide range of settings, both research and applied, where the detection and measurement of these learning effects may be of potential interest, such as those related to competence acquisition in developmental and educational contexts (e.g., Spada, 1977; Spada & McGaw, 1985) or to the substantive analysis of the learning processes that occur during a psychometric test (e.g., Lozano & Revuelta, 2020, 2021). Additionally, the presence of learning effects during the test may involve meaningful item associations beyond those explained by conventional item response models. In that case, assuming that the responses are locally independent would lead to incorrect parameter estimates and standard errors. Moreover, the inherent difficulty in distinguishing local dependence from multidimensionality (see Ip, 2010) may lead to overestimate the number of underlying factors when there are local dependencies between items due to learning effects. Incorporating previous practice into the models may allow for the detection and measurement of the learning effects as well as for the obtaining of unbiased estimates of item and person parameters while avoiding over-factoring.

A variety of models have been developed to account for the learning that takes place throughout a test (e.g., Deonovic et al., 2018; Fischer & Formann, 1982; Hohensinn et al., 2008; Kempf, 1977; Scheiblechner, 1972; Spada, 1977; Verguts & De Boeck, 2000; Verhelst & Glas, 1993). These models may be classified as contingent and non-contingent learning models (Verguts & De Boeck, 2000). Contingent learning models assume that learning depends on the correctness of the responses given to the items (e.g., Kempf, 1977; Verguts & De Boeck, 2000; Verhelst & Glas, 1993), whereas non-contingent learning models assume that learning occurs regardless of the correctness of the responses (e.g., Fischer & Formann, 1982; Scheiblechner, 1972; Spada, 1977). Another distinction can be made between descriptive and explanatory learning models (De Boeck & Wilson, 2004). Descriptive learning models are just aimed at measuring the learning effect, whereas explanatory learning models not only measure the learning effect but also explain it in terms of person and/or item properties. Most of the existing learning models are descriptive (e.g., Kempf, 1977; Verguts & De Boeck, 2000; Verhelst & Glas, 1993); however, a few models may be considered explanatory in that they account for the learning effect in terms of the operations involved in the items (e.g., Deonovic et al., 2018; Fischer & Formann, 1982; Scheiblechner, 1972; Spada, 1977). Interestingly, to date, all the explanatory learning models are non-contingent models and, therefore, do not make any distinction between correct and incorrect responses.

In the present paper, an explanatory contingent learning model is presented that is a generalization of the operation-specific learning model (OSLM) introduced by Scheiblechner (1972; see also Fischer & Formann, 1982; Spada, 1977). The OSLM accounts for the non-contingent learning that takes place during a psychometric test due to the repeated use of the cognitive operations required by the items. In the OSLM, the learning parameter is specific to each cognitive operation, and the learning component of the model is derived from the number of times the person has practiced in previous items each of the operations involved in the current item. The OSLM is subsumed by the proposed model, which accounts for the possibilities that learning may be derived from all the previous responses equally (non-contingent learning), from correct responses only (contingent learning), or from correct and incorrect responses in different degree (differential contingent learning). The distinction between correct and incorrect responses is reasonable in that learning is traditionally assumed to be greater when the examinee answers the items correctly. However, the reverse may also be true, since, according to the definition of learning implied in the OSLM (i.e., *a decrease in the difficulty associated with a specific cognitive operation throughout the test as a function of practice*), learning is potentially greater for those operations that are more difficult and, therefore, result in a greater number of incorrect responses at the beginning of the test.

In the next section, the new model is introduced and described in detail by discussing special cases subsumed by the general formulation. Model identification is described in Sect. 3. Section 4 describes a Bayesian framework for model estimation and evaluation. Section 5 includes a simulation study in which the performance of the estimation and evaluation methods is examined. Section 6 provides an empirical analysis to illustrate the applicability of the model to real data. Finally, a summary and concluding remarks are given in Sect. 7.

## Model Specification

The models presented in this paper are based on the Rasch model (Rasch, 1960). For a Rasch model, the logit of a correct response for person *i* ($i=1,2,\ldots ,I$) to item *j* ($j=1,2,\ldots ,J$) is given by:

1 $$\begin{aligned} {{\,\mathrm{logit}\,}}\left[ X_{ij}=1\right] =\theta _i-\beta _j, \end{aligned}$$

where $\theta _i$ is the ability of person *i*, and $\beta _j$ is the difficulty of item *j*. The linear logistic test model (LLTM; Fischer, 1973, 1983, 1995; Scheiblechner, 1972) decomposes the difficulty parameter of the Rasch model into a linear combination that represents the weighted sum of the difficulties of the cognitive operations involved in the item. That is:

2 $$\begin{aligned} {{\,\mathrm{logit}\,}}\left[ X_{ij}=1\right] =\theta _i-\sum _{m=1}^Mw_{jm}\alpha _m, \end{aligned}$$

where $\alpha _m$ is a basic parameter that represents the difficulty of operation *m* ($m=1,2,\ldots ,M$), and $w_{jm}$ is the weight of item *j* on operation *m*. The model is completed by $\mathbf{W}$, a $J\times M$ matrix that contains the weights ($w_{jm}$) of each of the *J* items on each of the *M* operations. Each weight is given by the number of times operation *m* is involved in the solution of item *j*. The LLTM may be considered a restricted version (in which all the learning parameters are constrained to zero) of each of the learning models presented in the following subsections.

### Operation-specific Learning Model

Based on the idea underlying the LLTM, Scheiblechner (1972; see also Fischer & Formann, 1982; Spada, 1977) introduced the OSLM. The OSLM is a non-contingent learning model; that is, it considers that learning is derived from both correctly and incorrectly answered items equally. According to this model, the logit of a correct response for person *i* to item *j* is a function of the person ability, the difficulty of the cognitive operations involved in the item, and the practice of said operations accumulated during previous items:

3 $$\begin{aligned} {{\,\mathrm{logit}\,}}\left[ X_{ij}=1\right] =\theta _i-\sum _{m=1}^Mw_{jm}\left( \alpha _m -\delta _m\sum _{k=1}^{j-1}w_{km}\right) , \end{aligned}$$

where $\delta _m$ is a practice parameter that represents the change in the difficulty of operation *m* that occurs each time the operation is practiced, and $w_{km}$ is the weight of the previous item $k\, (k=1,2,\ldots ,j-1)$ on operation *m*. In this model, $\alpha _m$ represents the initial difficulty of operation *m*, independently of the practice effect. As can be appreciated, the Rasch item parameter is decomposed into an initial-difficulty component ($\sum w_{jm}\alpha _m$), derived from the cognitive operations involved in solving the item, and a practice component ($\sum w_{jm}\delta _m\sum w_{km}$), derived from practicing said operations in previous items. Note that only when operation *m* is involved in both the previous item and the current item is the practice effect associated with operation *m* ($\delta _m$) subtracted from $\alpha _m$. A positive sign for the $\delta _m$ parameter implies a decrease in difficulty associated with operation *m* throughout the test as a function of practice, which may be interpreted as a learning effect. A negative sign, on the other hand, implies an increase in difficulty associated with operation *m* as a function of practice, which may be interpreted as fatigue or loss of attention. These fatigue effects associated with specific operations may occur, for example, in relatively easy operations that the subjects tend to perform correctly at the beginning of the test but that are prone to errors later on in the test due to the progressive effects of fatigue or loss of interest and/or attention. It should be noted that, although the OSLM models the effect of previous practice on the item response, like the LLTM and the Rasch model, it does not assume local dependence between items.

### Operation-specific Contingent Learning Model

In contrast to the OSLM, the operation-specific contingent learning model (OSCLM) assumes that the mere exposure to items does not contribute to learning. According to the OSCLM, learning takes place only when the items are answered correctly:

4 $$\begin{aligned} {{\,\mathrm{logit}\,}}\left[ X_{ij}=1\right] =\theta _i-\sum _{m=1}^Mw_{jm}\left( \alpha _m -\delta _m\sum _{k=1}^{j-1}x_{ik}w_{km}\right) , \end{aligned}$$

where $\delta _m$ represents the change in the difficulty of operation *m* that results from practicing the operation in a correctly answered item, and $x_{ik}$ is the response of person *i* to the previous item *k*. Note that only when $x_{ik}=1$ is the practice effect associated with operation $m\,(\delta _m)$ subtracted from $\alpha _m$. The contingent nature of the practice component implies that, unlike the OSLM, the OSCLM assumes local dependencies between items.

### Operation-specific Differential Contingent Learning Model

Finally, the operation-specific differential contingent learning model (OSDCLM) considers that learning takes place in both correctly and incorrectly answered items, although, unlike the OSLM, the amount of learning that is derived in both cases may differ:

5 $$\begin{aligned} {{\,\mathrm{logit}\,}}\left[ X_{ij}=1\right] =\theta _i-\sum _{m=1}^Mw_{jm}\left[ \alpha _m -\delta _m\sum _{k=1}^{j-1}x_{ik}w_{km}-\gamma _m\sum _{k=1}^{j-1}(1-x_{ik})w_{km}\right] , \end{aligned}$$

where $\gamma _m$ is a practice parameter that represents the change in the difficulty of operation *m* that results from practicing the operation in an incorrectly answered item. Note that when $x_{ik}=0$, it is $\gamma _m$ and not $\delta _m$ that is subtracted from $\alpha _m$. A positive sign for the $\gamma _m$ parameter indicates that even when an item involving operation *m* is incorrectly answered, the difficulty of that operation decreases in subsequent items. This may be due to the fact that many participants perform operation *m* correctly (and, therefore, some amount of learning is derived from practicing the operation), but they fail to perform other operations involved in the item and, consequently, answer the item incorrectly. Alternatively, the positive sign may be due to the fact that, for many participants, operation *m* requires successive approximations over several items in order for it to be properly performed. A negative sign, on the other hand, indicates that answering incorrectly an item involving operation *m* increases the difficulty of that operation in subsequent items, which may be attributed to fatigue or loss of interest and/or attention. The OSDCLM generalizes both the OSLM and the OSCLM. In this regard, the OSCLM is a restricted OSDCLM in which all $\gamma _m=0$, whereas the OSLM is a restricted OSDCLM in which $\delta _m=\gamma _m$ for each *m*.

## Model Identification

In the LLTM, for the basic parameters ($\alpha _m$) to be estimated by means of conditional maximum likelihood (CML), the matrix $\mathbf{W}^+=(\mathbf{W};\mathbf{1})$ (i.e., $\mathbf{W}$ supplemented with a column vector of ones) must have full column rank; that is, $rank(\mathbf{W}^+)=M+1$ (Fischer, 1983). As a result, the number of operations is restricted to $M\le J-1$. The full column rank condition of $\mathbf{W}^+$ ensures that the Rasch item parameters ($\beta _j$) can be decomposed uniquely into the LLTM basic parameters ($\alpha _m$) while fixes the scale of the latent variable ($\theta _i$). In Bayesian inference, by contrast, the $\theta$ scale is fixed by specifying the prior distribution of the parameter, so the looser condition of full column rank of $\mathbf{W}$, $rank(\mathbf{W})=M$, is enough to ensure the uniqueness of the relation between the parameters of the Rasch model and the LLTM. Consequently, in Bayesian inference, the original restriction $M\le J-1$ is relaxed to $M\le J$.

Mathematically, the OSLM is an LLTM with weigh matrix $\mathbf{Q}=(\mathbf{W};\mathbf{V})$, where $\mathbf{V}$ is a $J\times M$ matrix whose elements represent previous practice. More specifically, the elements in $\mathbf{V}$ are given by:

6 $$\begin{aligned} v_{jm}=w_{jm}\sum _{k=1}^{j-1}w_{km}. \end{aligned}$$

Therefore, in the OSLM, the full column rank condition for CML estimation is ${ rank}(\mathbf {Q}^+)=2M+1$, and the number of operations is restricted to $M\le (J-1)/2$. In Bayesian inference, these restrictions are relaxed to ${ rank}(\mathbf{Q})=2M$ and $M\le J/2$.

In the OSCLM and the OSDCLM, the weigh matrices are $\mathbf{Q}=(\mathbf{W};\mathbf{V}_t)$ and $\mathbf{Q}=(\mathbf{W};\mathbf{V}_t;\mathbf{U}_t)$, respectively, where $\mathbf{V}_t$ and $\mathbf{U}_t$ are $J\times M$ matrices whose elements represent the amount of correct and incorrect previous practice for each item and operation. Specifically, the elements in $\mathbf{V}_t$ and $\mathbf{U}_t$ are given by:

7 $$\begin{aligned} \begin{aligned} v_{tjm}&=w_{jm}\sum _{k=1}^{j-1}x_{tk}w_{km} \text{ and }\\ u_{tjm}&=w_{jm}\sum _{k=1}^{j-1}(1-x_{tk})w_{km}, \end{aligned} \end{aligned}$$

where $t\,(t=1,2,\ldots ,T)$ denotes a specific response pattern, and $T=2^J$ is the number of different response patterns.

Let ${{\varvec{x}}}_t'=(x_{t1},x_{t2},\ldots ,x_{tJ})$ be a vector of responses to the *J* items. Assuming that $\theta$ is a random effect that follows a standard normal distribution, the marginal probability of ${{\varvec{x}}}_t$ is:

8 $$\begin{aligned} p_t=\int _{-\infty }^{\infty }\frac{\exp (\lambda _t)}{\sum _{h=1}^T \exp (\lambda _h)}f(\theta )d\theta , \end{aligned}$$

where $\lambda _t$ is a parameter associated with response pattern *t*, and $f(\theta )$ is the standard normal density function. The OSCLM and the OSDCLM impose the following structure on the parameters:

9 $$\begin{aligned} \lambda _t=s_t\theta +{{\varvec{r}}}_t'\varvec{\xi }, \end{aligned}$$

where $s_t=\sum _{j=1}^J x_{tj}$ is the number-right score of response pattern *t*, ${{\varvec{r}}}_t'$ is a row-vector of coefficients associated with response pattern *t*^1^, and ${\varvec{\xi }}$ is the vector of structural parameters^2^. The OSCLM and the OSDCLM assume that the vector of $\lambda _t$ parameters, ${\varvec{\lambda }}=(\lambda _t)_{t=1}^T$, is:

10 $$\begin{aligned} {\varvec{\lambda }}={{\varvec{s}}}\theta +\mathbf{R}{{\varvec{\xi }}}, \end{aligned}$$

where ${{\varvec{s}}}=(s_t)_{t=1}^T$ is the vector of number-right scores of the *T* response patterns, and $\mathbf{R}$ is a matrix of coefficients whose rows are the vectors ${{\varvec{r}}}_t'={{\varvec{r}}}_1',{{\varvec{r}}}_2',\ldots ,{{\varvec{r}}}_T'$.

The analysis of the identifiability of ${{\varvec{\xi }}}$ is based on the Jacobian matrix (Bishop et al., 2007; Cox, 1984):

11 $$\begin{aligned} \mathbf{J}=\frac{\partial }{\partial {{\varvec{\xi }}}}\log {{\varvec{p}}} =(\mathbf{I}-\mathbf{1}{{\varvec{p}}}')\mathbf{R}, \end{aligned}$$

where $\mathbf{I}$ is an identity matrix of order *T*, $\mathbf{1}$ is a vector of ones, and ${{\varvec{p}}}=(p_t)_{t=1}^T$ is the vector of probabilities of the *T* response patterns. The vector ${{\varvec{\xi }}}$ is identifiable if $\mathbf{J}$ has full column rank. The matrix $(\mathbf{I}-\mathbf{1}{{\varvec{p}}}')$ is deficient in rank (it has rank $T-1$) because the elements in ${\varvec{p}}$ are constrained to sum 1. Specifically, $(\mathbf{I}-\mathbf{1}{{\varvec{p}}}')\mathbf{1}=\mathbf{0}$. Therefore, if the vector $\mathbf{1}$ were in the column space of $\mathbf{R}$, there would be a vector ${\varvec{\tau }}$ such that $(\mathbf{I}-\mathbf{1}{{\varvec{p}}}')\mathbf{R}{\varvec{\tau }}=\mathbf{0}$, and $\mathbf{J}$ would be deficient in rank. Moreover, from the theory of multinomial maximum likelihood estimation, the information matrix for ${\varvec{\xi }}$ can be computed from the Jacobian matrix by the equation (Revuelta, 2012):

12 $$\begin{aligned} {\mathcal {I}}=\mathbf{J}'{} \mathbf{D}{} \mathbf{J}, \end{aligned}$$

where $\mathbf{D}=\text{ diag }({{\varvec{p}}})$. If $\mathbf{J}$ were deficient in rank, ${\mathcal {I}}$ would be so. Consequently, the identifiability condition for ${\varvec{\xi }}$ is that the matrix $\mathbf{R}^+=(\mathbf{R};\mathbf{1})$ has full column rank. In practice, the analysis of empirical identifiability is based on the response patterns that have been actually realized in the sample. Let $\hat{\mathbf{R}}^+$ be the matrix of coefficients based on the realized response patterns. The full column rank of $\hat{\mathbf{R}}^+$ is necessary for the observed information matrix to be of full rank. However, since $\hat{\mathbf{R}}^+$ has size $N \times 3M$, where *N* can be in the order of hundreds or thousands, it is more computationally convenient to verify the equivalent condition that the matrix ${\hat{\mathbf{R}}}^{+'}{\hat{\mathbf{R}}}^+$ has full rank.

## Bayesian Framework

A Bayesian framework is presented for the estimation and evaluation of the proposed model. In this work, Bayesian methods were implemented by means of Markov chain Monte Carlo (MCMC) simulation (Brooks et al., 2011). Applications of Bayesian MCMC in the field of item response modeling can be seen in Fox (2010) and Levy and Mislevy (2016).

### Model Estimation

In Bayesian analysis, MCMC routines are usually employed to derive an empirical approximation to the posterior distribution of the parameters. In the present work, MCMC simulation was run using Stan (Carpenter et al., 2017; Gelman et al., 2015). Stan is a programming software that implements the no-U-turn sampler (NUTS; Hoffman & Gelman, 2014), an extension of the Hamiltonian Monte Carlo (HMC; Duane et al., 1987; Neal, 1994, 2011) algorithm. HMC overcomes some of the limitations of the traditional Gibbs sampler (Geman & Geman, 1984) and the Metropolis algorithm (Metropolis et al., 1953), particularly in terms of computational efficiency in exploring the posterior parameter space (Gelman et al., 2013).

### Model Evaluation

In the Bayesian context, model assessment is typically based on posterior predictive model checking (PPMC; Gelman et al., 1996). PPMC is conducted based on discrepancy measures that are intended to capture relevant features of the data. The realized values of the model-data discrepancy, $D({\mathbf {X}};{\varvec{\theta }},{\varvec{\xi }})$ (where ${\varvec{\theta }}$ and ${\varvec{\xi }}$ represent the vectors of incidental and structural parameters, respectively), are compared to those obtained from the posterior predictive distribution, $D({\mathbf {X}}^{\mathrm {rep}};{\varvec{\theta }},{\varvec{\xi }})$ (where *rep* stands for *replicated* data). The results are summarized by means of the posterior predictive *p* value (PPP value; Gelman et al., 1996; Meng, 1994), the tail-area probability of the realized value of the discrepancy under the posterior predictive distribution of the discrepancy measure:

13 $$\begin{aligned} {\mathrm {PPP}}=P\left[ D\left( {\mathbf {X}}^{\mathrm {rep}};{\varvec{\theta }},{\varvec{\xi }}\right) \ge D\left( {\mathbf {X}};{\varvec{\theta }},{\varvec{\xi }}\right) \mid {\mathbf {X}}\right] . \end{aligned}$$

In the present study, the discrepancy between the data and the model was estimated via two discrepancy statistics: the odds-ratio (OR; Chen & Thissen, 1997; Sinharay, 2005) and the Bayesian latent residual (BLR; Albert & Chib, 1995; Fox, 2010). The OR is a measure of association between pairs of items that is computationally simple and does not depend on the fitted model. The OR for items *j* and $j'$ is defined as:

14 $$\begin{aligned} \hbox {OR}_{jj'}=\frac{n_{11}n_{00}}{n_{10}n_{01}}, \end{aligned}$$

where $n_{xx'}$ is the number of individuals scoring *x* on item *j* and $x'$ on item $j'$. The OR is useful for identifying inter-item associations beyond those explained by the model. Given that practice effects may elicit local dependencies between items, the OR is potentially useful for detecting the presence of learning effects during the test. Measures of inter-item associations at the item level and at the test level are obtained by summing the OR values over the pairs of items.

The BLR is a measure of overall fit that is not specifically tied to local dependencies. The BLR is based on an augmented (latent) data approach and is defined as the difference between the latent response and the expected response according to the model. For instance, for a Rasch model, the BLR corresponding to observation $X_{ij}$ is defined as:

15 $$\begin{aligned} \varepsilon _{ij}=Z_{ij}-\theta _i+\beta _j, \end{aligned}$$

where $Z_{ij}$ is the latent response of person *i* to item *j*, which, conditional on person and item parameters, follows a logistic distribution with expected value given by ${{\,\mathrm{logit}\,}}\left[ X_{ij}=1\right]$. Computational formulas for the BLR are given in Fox (2010). The squared residuals can be summed over individuals to obtain an item-specific discrepancy statistic. A global measure of fit at the test level is obtained by summing the values of the squared residuals over the items.

The PPP value is the proportion of draws in which the posterior predictive value of the discrepancy statistic is equal to or higher than the realized value. PPP values close to .5 indicate that the realized value is in the middle of the posterior predictive distribution of the discrepancy, evidencing adequate data-model fit; whereas extreme PPP values, close to zero or one, indicate that the realized value is in the upper or lower tail of the distribution, respectively, evidencing that the model is underpredicting or overpredicting the features captured by the discrepancy statistic. For instance, in the case of the OR, PPP values close to zero (one) indicate that the observed data exhibit more (less) local dependence than expected based on the model.

### Model Comparison and Selection

Complementarily, other methods can be used for model comparison and selection: the widely applicable information criterion (WAIC; Watanabe, 2010, 2013) and the leave-one-out cross validation (LOO; Gelman et al., 2014). These methods quantify the out-of-sample predictive performance of competing models using the log-likelihood evaluated at the posterior simulations of the parameter values. WAIC and LOO adjust the log pointwise predictive density (lpd) of the observed data by penalizing for model complexity based on the effective number of parameters. Such penalty allows for the prevention of the over-fitting exhibited by more complex models by virtue of their higher flexibility.

Let $l\,(l=1,2,\ldots ,L)$ be a draw from the posterior distribution. In the case of WAIC, the estimated expected log pointwise predictive density (elpd) is given by (Vehtari et al., 2016):

16 $$\begin{aligned} {\widehat{\mathrm {elpd}}_{\mathrm {waic}}}={\widehat{\mathrm {lpd}}}-{\widehat{p}}_{\mathrm {waic}}, \end{aligned}$$

where ${\widehat{\mathrm {lpd}}}$ is the computed log pointwise predictive density:

17 $$\begin{aligned} {\widehat{\mathrm {lpd}}}=\sum _{i=1}^I\sum _{j=1}^J\log \left[ \frac{1}{L} \sum _{l=1}^Lp\left( x_{ij}\mid {\varvec{\theta }}^l,{{\varvec{\xi }}}^l\right) \right] , \end{aligned}$$

and ${\widehat{p}}_{\mathrm{waic}}$ is the estimated effective number of parameters, which can be obtained based on the posterior variance of the log predictive density for each data point $x_{ij}$:

18 $$\begin{aligned} {\widehat{p}}_{\mathrm{waic}}=\sum _{i=1}^I\sum _{j=1}^JVar_{l=1}^L \left[ \log p\left( x_{ij}\mid {\varvec{\theta }}^l,{{\varvec{\xi }}}^l\right) \right] . \end{aligned}$$

The ${\widehat{\mathrm {elpd}}_{\mathrm{waic}}}$ is usually converted to deviance scale as follows:

19 $$\begin{aligned} {\mathrm {WAIC}}=-2{\widehat{\mathrm {elpd}}_{\mathrm{waic}}}. \end{aligned}$$

In the case of LOO, the estimated elpd, obtained by Pareto smoothed importance sampling, is given by (Vehtari et al., 2016):

20 $$\begin{aligned} {\widehat{\mathrm {elpd}}_{\mathrm{loo}}}=\sum _{i=1}^I\sum _{j=1}^J\log \left[ \frac{\sum _{l=1}^Lw_{ij}^lp\left( x_{ij}\mid {\varvec{\theta }}^l,{{\varvec{\xi }}}^l \right) }{\sum _{l=1}^Lw_{ij}^l}\right] , \end{aligned}$$

where $w_{ij}^l$ is a vector of smoothed weights for each data point $x_{ij}$. For LOO, the effective number of parameters is given by:

21 $$\begin{aligned} {\widehat{p}}_{\mathrm{loo}}={\widehat{\mathrm {lpd}}}-{\widehat{\mathrm {elpd}}_{\mathrm{loo}}}. \end{aligned}$$

The LOO information criterion (LOOIC), expressed on the deviance scale, is defined as:

22 $$\begin{aligned} {\mathrm {LOOIC}}=-2{\widehat{\mathrm {elpd}}_{\mathrm{loo}}}. \end{aligned}$$

Lower values of WAIC and LOOIC indicate higher predictive accuracy. Compared to PPMC, WAIC and LOO have the advantage of avoiding re-sampling and, therefore, are less computationally intensive. However, WAIC and LOO are not intended to test a hypothesis of model fit but to compare models in order to select the one that fits the data best. In the present work, PPMC was used for model evaluation, whereas WAIC and LOO were used complementarily for model comparison and selection.

## Simulation Study

A simulation study was conducted to test whether the Bayesian estimation and model evaluation methods allow for the recovery of the true item parameters and the identification of the model used to generate the data, respectively. Particular attention was paid to examine the bias of the estimates when there were learning effects in the data that were not taken into account in the model.

### Method

In order to study different conditions of misspecification, a $4\times 5$ factorial design was used for the simulation study, resulting from the combination of generating models and fitted models (the OSDCLM, OSCLM, OSLM, and LLTM were used as generating models, while the same models plus the Rasch model were used as fitted models).

One hundred data sets of dichotomous responses were simulated from each generating model. The simulation was conducted with R version 3.6.1 (R Development Core Team, 2019). The sample size, test length, weight matrix, and true values of the structural parameters ($\alpha _m$, $\delta _m$, and $\gamma _m$) were taken from the empirical study described in Sect. 6 (the weight matrix is shown in Table 4, and the structural parameters are shown in Table 9). The true values of the incidental parameters ($\theta _i$) were generated from a standard normal distribution.

The models were estimated from each simulated data set using the RStan R package version 2.19.2 (Stan Development Team, 2019). Four Markov chains of 2,000 samples each were run. The first half of the samples were discarded as burn-in, and the remaining samples were used to estimate the Bayesian posterior probabilities. The potential scale reduction statistic (Gelman & Rubin, 1992) was used to evaluate the convergence of parameter estimates. A weakly informative prior, *N*(0, 100), was used for all structural parameters, whereas a standard normal distribution was used as prior for the incidental parameters.

To assess the fit of the models to the data, a sample of predicted responses was generated for each sample of simulated parameters, and the PPP value (Gelman et al., 1996; Meng, 1994) was computed based on the discrepancy measures, OR (Chen & Thissen, 1997; Sinharay, 2005) and BLR (Albert & Chib, 1995; Fox, 2010), at the test level. The hypothesis that the model fits the data was rejected when the PPP value was less than .05 or greater than .95. The performance of the discrepancy measures was assessed by the average PPP value over the 100 simulated samples as well as by the empirical proportion of rejections (EPR), that is, the proportion of simulated samples in which the fitted model is rejected. When the fitted model coincides with the model used to generate the data, the EPR is an estimate of the false-positive error rate of the test, whereas when the fitted model and the generating model do not coincide, the EPR is an estimate of the sensitivity of the test.

Additionally, two information criterion measures were obtained using the loo R package (Vehtari et al., 2016): WAIC (Watanabe, 2010, 2013) and LOOIC (Gelman et al., 2014). As described above, these measures quantify the discrepancy between the model and the data while taking into account model complexity. They are not intended to test a hypothesis of model fit but to select the best model from a number of competing models. Lower values of WAIC and LOOIC indicate better balance between fit and parsimony. In this study, for each simulated sample, WAIC and LOOIC were used to select the best model from among the fitted models. For each condition of the study, the performance of WAIC and LOOIC was assessed by their average value over the simulated samples as well as by the empirical proportion of selections (EPS), that is, the proportion of simulated samples in which the fitted model is selected.

Item parameter recovery was assessed using measures of precision, bias, and accuracy of the estimation procedure. The standard error (SE) of the estimate was used as a measure of statistical variability (precision) of the estimation procedure. For instance, the SE for the $\alpha$ parameter is defined as:

23 $$\begin{aligned} {\mathrm {SE}}\left( {\hat{\alpha }}\right) =\sqrt{\frac{1}{N-1}\sum _{n=1}^N \left[ \frac{\displaystyle \sum _{m=1}^M\left( {\hat{\alpha }}_{nm} -\overline{{\hat{\alpha }}}_{m}\right) ^2}{M}\right] }, \end{aligned}$$

where $n\,(n=1,2,\ldots ,N)$ denotes a simulated sample, *N* is the number of simulated samples (in this study, $N=100$), *M* is the number of $\alpha$ parameters, ${\hat{\alpha }}_{nm}$ is the EAP estimate of the *m*-th $\alpha$ parameter in sample *n*, and $\overline{{\hat{\alpha }}}_m$ is the mean of the estimates of $\alpha _m$ over the *N* samples. Unlike bias and accuracy, precision depends only on the estimates (it does not depend on the true value of the parameter).

The bias quantifies the difference between the mean of the parameter estimates over the *N* samples and the true value of the parameter. The absolute bias for the $\alpha$ parameter is defined as:

24 $$\begin{aligned} {\mathrm {Bias}}\left( {\hat{\alpha }}\right) =\frac{\displaystyle \sum _{m=1}^M \left| \overline{{\hat{\alpha }}}_m-\alpha _m\right| }{M}, \end{aligned}$$

where $\alpha _m$ is the true value of the *m*-th $\alpha$ parameter.

The root-mean-square error (RMSE) combines precision and bias to provide a measure of accuracy in parameter recovery. The RMSE quantifies the average difference between the true and the estimated parameters over the *N* samples. The RMSE for the $\alpha$ parameter is defined as:

25 $$\begin{aligned} {\mathrm {RMSE}}\left( {\hat{\alpha }}\right) =\frac{1}{N}\sum _{n=1}^N \sqrt{\frac{\displaystyle \sum _{m=1}^M\left( {\hat{\alpha }}_{nm}-\alpha _m\right) ^2}{M}}. \end{aligned}$$

The SE, bias, and RMSE for the $\delta$ and $\gamma$ parameters are defined in the same way.

### Results

Table 1 shows the mean PPP value and the EPR of the discrepancy measures for each combination of generating model and fitted model. As expected, for each generating model, fitting the true or a more general model led to a mean PPP value close to .5, indicating good model-data fit. On the contrary, fitting a more restrictive model than the one used to generate the data led to an extreme mean PPP value, close to zero or one, indicating model misfit. Likewise, when the true or a more general model was fitted to the data, the EPR was close to zero, indicating a low false-positive error rate. However, fitting a more restrictive model led to an EPR close to one, revealing the high sensitivity of the procedure in the detection of the different types of learning. The above applies to all conditions except when there were non-contingent learning effects in the data (i.e., when the OSLM was the generating model) and the estimated model was the LLTM. In that condition, the BLR and, to a lesser extent, the OR showed low sensitivity. It is also worth noting the low EPR values associated with the Rasch model when the data were generated with the OSLM. This result was due to the fact that the OSLM, like the LLTM, is a restricted Rasch model that does not model local dependencies between items. Consequently, as a more general model, the Rasch model is expected to fit data generated with the OSLM.

**Table 1:** Average posterior predictive *p*-value ($\overline{\mathrm {PPP}}$) and empirical proportion of rejections (EPR) of the discrepancy statistics for each combination of generating model and fitted model

Generating model	Discrepancy statistic	Fitted model									
		OSDCLM		OSCLM		OSLM		LLTM		Rasch	
		$\overline{\mathrm {PPP}}$	EPR	$\overline{\mathrm {PPP}}$	EPR	$\overline{\mathrm {PPP}}$	EPR	$\overline{\mathrm {PPP}}$	EPR	$\overline{\mathrm {PPP}}$	EPR
OSDCLM	OR	.531	.00	.000	1.00	.000	1.00	.000	1.00	.000	1.00
	BLR	.420	.00	1.000	1.00	1.000	1.00	1.000	1.00	1.000	1.00
OSCLM	OR	.546	.00	.526	.00	.000	1.00	.000	1.00	.000	1.00
	BLR	.413	.00	.441	.00	1.000	1.00	1.000	1.00	.999	1.00
OSLM	OR	.530	.00	1.000	1.00	.485	.02	.068	.68	.492	.03
	BLR	.412	.00	.013	.99	.446	.00	.231	.00	.415	.00
LLTM	OR	.547	.00	.517	.02	.512	.02	.506	.02	.517	.01
	BLR	.418	.00	.455	.00	.446	.00	.474	.00	.421	.00

Table 2 shows the mean values of WAIC and LOOIC and their corresponding EPS for each combination of generating model and fitted model. Based on both WAIC and LOOIC, for each condition of generating model, the true model (followed by more general models) led to the lowest mean discrepancy between the data and the model as well as to the highest EPS.

**Table 2:** Average WAIC and LOOIC (Mean) and empirical proportion of selections (EPS) for each combination of generating model and fitted model

Generating model	Comparison index	Fitted model									
		OSDCLM		OSCLM		OSLM		LLTM		Rasch	
		Mean	EPS	Mean	EPS	Mean	EPS	Mean	EPS	Mean	EPS
OSDCLM	WAIC	7104.653	1.00	7215.236	.00	7268.778	.00	7411.123	.00	7270.365	.00
	LOOIC	7108.544	1.00	7218.581	.00	7272.001	.00	7414.283	.00	7273.663	.00
OSCLM	WAIC	7187.204	.35	7182.073	.65	7464.165	.00	7618.335	.00	7465.728	.00
	LOOIC	7190.972	.35	7185.760	.65	7467.622	.00	7621.753	.00	7469.246	.00
OSLM	WAIC	7345.984	.37	7694.434	.00	7340.335	.60	8075.063	.00	7345.466	.03
	LOOIC	7350.293	.37	7698.744	.00	7344.569	.60	8079.357	.00	7349.747	.03
LLTM	WAIC	7619.407	.20	7613.068	.29	7614.895	.04	7610.255	.45	7620.780	.02
	LOOIC	7622.836	.20	7616.405	.29	7618.245	.04	7613.508	.45	7624.205	.02

Table 3 shows the SE, bias, and RMSE for each combination of estimated parameter, generating model, and fitted model. For each generating model, the SE was minimized by the most restrictive model (the LLTM, OSCLM, and OSDCLM, for the $\alpha$, $\delta$, and $\gamma$ parameters, respectively), whereas the bias was minimized by the true or a more general model. As expected, for each generating model, fitting the true model minimized the RMSE and, therefore, maximized the accuracy of the estimates. Conversely, fitting a more restrictive model than the one used to generate the data led to inaccurate estimates of the difficulty and practice parameters. In order to rule out potential differential effects associated with the sign of the parameter, the SE, bias, and RMSE were also obtained for each operation separately without evidence of differential effects.

**Table 3:** Standard error (SE), bias, and root-mean-square error (RMSE) for each combination of estimated parameter, generating model, and fitted model

Estimated parameter	Generating model	Statistic	Fitted model			
			OSDCLM	OSCLM	OSLM	LLTM
$\alpha$	OSDCLM	SE	0.487	0.120	0.352	0.083
		Bias	0.060	1.753	1.129	1.716
		RMSE	0.413	2.398	1.610	2.054
	OSCLM	SE	0.488	0.121	0.401	0.085
		Bias	0.008	0.009	0.340	1.072
		RMSE	0.406	0.113	0.487	1.190
	OSLM	SE	0.473	0.115	0.476	0.074
		Bias	0.008	1.639	0.010	1.731
		RMSE	0.412	1.943	0.411	1.925
	LLTM	SE	0.516	0.125	0.491	0.103
		Bias	0.040	0.010	0.004	0.016
		RMSE	0.430	0.115	0.407	0.096
$\delta$	OSDCLM	SE	0.134	0.068	0.089	
		Bias	0.014	0.523	0.407	
		RMSE	0.121	0.713	0.435	
	OSCLM	SE	0.130	0.074	0.100	
		Bias	0.008	0.008	0.319	
		RMSE	0.116	0.069	0.354	
	OSLM	SE	0.136	0.075	0.129	
		Bias	0.007	0.404	0.005	
		RMSE	0.125	0.480	0.116	
$\gamma$	OSDCLM	SE	0.140			
		Bias	0.021			
		RMSE	0.125			

### Conclusions

The simulation study illustrates the good performance of PPMC for model evaluation and selection as well as the accuracy of the MCMC algorithm in recovering the true parameters from simulated data. Regarding model evaluation, PPMC based on the discrepancy statistics showed good performance in identifying learning effects in the data. Specifically, the OR and BLR statistics only showed low sensitivity in one condition. Additionally, WAIC and LOO demonstrated relatively good performance in model comparison and selection, although they showed a certain tendency to favor complex models. Based on these results, when sufficient computational resources are available, the use of PPMC should be preferred also for model comparison and selection, taking as a decision rule to select the simplest model that shows an acceptable fit to the data. Regarding parameter recovery, as expected, fitting the true model provided the most accurate parameter estimates. On the contrary, when there were learning effects in the data that were not taken into consideration in the model formulation, the resulting parameter estimates were considerably inaccurate.

## Empirical Study

An empirical study was conducted to illustrate the performance and applicability of the proposed framework for detecting practice effects in real data. Specifically, the models were fitted to data from a fraction arithmetic test (Tatsuoka, 1984) whose items are based on several arithmetic operations that are repeatedly applied throughout the test.

### Method

The data consists of responses to 15 items involving subtraction of fractions by 536 examinees. The data set was originally used by Tatsuoka (1984) and is included in the CDM R package (George et al., 2016). The matrix $\mathbf{W}$ used in this study was defined by de la Torre (2009) in the context of cognitive diagnosis modeling (see Table 4). In this example, the matrix $\hat{\mathbf{R}}^+$ satisfies the rank condition, $rank(\hat{\mathbf{R}}^+)=16$, and, consequently, the vector ${\varvec{\xi }}$ is empirically identified.

The same models, estimation method, and model evaluation procedures tested in the simulation study were used with the empirical data. A prior sensitivity study was conducted to investigate the effect of prior choice on the posterior parameter estimates. A normal prior distribution was used with mean set equal to zero, while the value of the variance was manipulated across conditions (i.e., 1, 5, 10, 50, 100, 500, 1,000, 5,000, and 1,000,000).

**Table 4:** Transposed weight matrix for the fraction-subtraction items (de la Torre, 2009)

Operation	Item														
	1	2	3	4	5	6	7	8	9	10	11	12	13	14	15
1	1	1	1	1	0	1	1	1	1	1	1	1	1	1	1
2	0	1	0	1	0	1	1	1	0	0	0	0	1	1	1
3	0	1	0	1	1	1	1	0	1	1	1	1	1	1	1
4	0	1	0	1	0	1	1	0	0	1	0	1	1	1	1
5	0	0	0	1	0	0	0	0	0	1	0	0	0	1	0

### Results

The prior sensitivity analysis revealed that the posterior parameter estimates were robust to different prior distributions. More specifically, the average standard deviations for the $\alpha$, $\delta$, and $\gamma$ parameter estimates were .168, .042, and .040, respectively. Moreover, when removing the estimates corresponding to *N*(0, 1), the average standard deviations were .017, .004, and .004, whereas when removing the estimates corresponding to *N*(0, 1) and *N*(0, 5), the average standard deviations were .010, .003, and .003. The results shown in this section were obtained by using a weakly informative prior, *N*(0, 100), for all structural parameters and a standard normal distribution for the incidental parameters.

Table 5 shows the model evaluation statistics at the test level for each of the fitted models. The PPP values of the discrepancy measures led to the rejection of the hypothesis of fit for the LLTM and the Rasch model in all cases (PPP $< .05$ or PPP $> .95$). More specifically, the observed and simulated values of the OR indicated that the data showed more local dependence than would be expected based on these models. According to the OR, the OSDCLM was the only model that reproduced the local dependencies present in the data ($\hbox {PPP} = .200$). Similarly, the PPP value of the BLR suggested that the OSDCLM was the only model that fitted the data well ($\hbox {PPP} = .467$).

**Table 5:** Model evaluation statistics at the test level for the fitted models

Fitted model	OR			BLR		
	Observed	Simulated(Sd)	PPP	Observed(Sd)	Simulated(Sd)	PPP
OSDCLM	1410.08	1272.77(175.13)	.200	4624.28(114.26)	4605.08(160.77)	.467
OSCLM	1410.08	570.21(45.57)	.000	3996.27(85.43)	5028.52(141.22)	1.000
OSLM	1410.08	410.76(23.36)	.000	3524.87(57.06)	5162.57(126.97)	1.000
LLTM	1410.08	403.21(22.49)	.000	3605.48(56.42)	5185.35(127.74)	1.000
Rasch	1410.08	419.13(24.30)	.000	3457.04(56.30)	5139.13(129.42)	1.000

Tables 6 and 7, respectively, show the OR and BLR statistics at the item level for the fitted models. Based on the PPP value of both the OR and the BLR, the OSDCLM was the model that fitted the data best, showing the lowest proportion of non-fitting items (PPP $< .05$ or PPP $> .95$).

**Table 6:** Odds-ratio at the item level for the fitted models

Item	Observed	OSDCLM		OSCLM		OSLM		LLTM		Rasch	
		Simulated(Sd)	PPP	Simulated(Sd)	PPP	Simulated(Sd)	PPP	Simulated(Sd)	PPP	Simulated(Sd)	PPP
1	184.13	85.79(13.86)	.000	71.85(8.53)	.000	54.17(5.47)	.000	53.88(5.60)	.000	54.87(5.50)	.000
2	111.53	89.67(13.17)	.062	66.75(7.93)	.000	54.08(5.24)	.000	53.32(5.27)	.000	55.12(5.43)	.000
3	201.61	120.94(30.48)	.022	84.72(11.24)	.000	54.85(5.92)	.000	53.82(5.70)	.000	57.90(7.75)	.000
4	174.98	133.93(22.88)	.050	75.87(9.72)	.000	54.78(5.56)	.000	53.82(5.49)	.000	55.69(5.80)	.000
5	45.50	29.09(4.48)	.002	26.37(3.29)	.000	54.29(5.45)	.960	53.11(5.14)	.941	55.20(5.72)	.964
6	199.64	119.51(18.62)	.000	68.73(8.35)	.000	54.40(5.43)	.000	53.40(5.23)	.000	56.14(6.04)	.000
7	258.48	136.47(22.12)	.000	70.16(8.49)	.000	54.28(5.28)	.000	53.37(5.29)	.000	55.18(5.47)	.000
8	154.30	249.75(78.48)	.932	81.04(14.00)	.000	55.75(6.52)	.000	53.84(5.61)	.000	56.47(6.55)	.000
9	204.39	159.63(48.81)	.154	54.10(8.28)	.000	55.79(6.61)	.000	54.81(6.16)	.000	56.23(6.19)	.000
10	193.02	224.32(46.90)	.743	109.51(13.79)	.000	54.51(5.45)	.000	53.95(5.40)	.000	55.48(5.59)	.000
11	147.40	161.75(43.40)	.580	48.16(7.24)	.000	54.64(5.70)	.000	54.76(6.30)	.000	55.68(5.98)	.000
12	251.13	195.70(36.18)	.072	108.95(13.32)	.000	54.18(5.24)	.000	53.39(5.23)	.000	55.51(5.70)	.000
13	170.65	227.53(45.68)	.932	86.04(10.40)	.000	54.84(5.60)	.000	53.40(5.29)	.000	55.36(5.40)	.000
14	251.01	332.97(68.58)	.907	98.15(12.95)	.000	56.02(6.39)	.000	54.09(5.57)	.000	57.38(6.72)	.000
15	272.40	278.47(58.14)	.479	90.02(11.12)	.000	54.94(5.66)	.000	53.44(5.33)	.000	56.04(5.93)	.000

**Table 7:** Bayesian latent residuals at the item level for the fitted models

Item	OSDCLM			OSCLM			OSLM		
	Observed(Sd)	Simulated(Sd)	PPP	Observed(Sd)	Simulated(Sd)	PPP	Observed(Sd)	Simulated(Sd)	PPP
1	314.48(20.09)	355.98(25.61)	.899	278.42(14.10)	353.49(29.27)	.991	253.41(17.98)	345.84(33.31)	.993
2	389.74(19.56)	355.67(26.27)	.147	472.92(27.45)	348.82(30.28)	.002	350.96(18.65)	355.52(30.23)	.549
3	206.30(13.42)	333.98(36.14)	1.000	240.25(15.74)	336.66(34.28)	.997	180.76(10.64)	333.83(35.81)	1.000
4	264.49(13.83)	342.73(32.43)	.990	246.32(16.00)	333.21(33.89)	.990	212.86(11.15)	350.64(31.54)	1.000
5	315.34(17.82)	356.98(24.70)	.915	278.77(13.64)	356.36(24.84)	.997	469.25(20.33)	344.60(32.94)	.001
6	258.84(16.90)	343.06(33.82)	.989	199.20(11.02)	346.05(32.18)	1.000	200.90(12.10)	354.02(31.22)	1.000
7	236.64(16.08)	335.15(36.75)	.994	203.48(12.18)	346.36(33.10)	1.000	186.39(10.75)	354.07(30.48)	1.000
8	400.41(35.46)	272.97(42.10)	.017	300.53(24.34)	312.95(39.41)	.600	261.64(21.50)	320.29(37.74)	.904
9	279.99(21.73)	294.49(42.47)	.606	229.42(17.34)	329.76(38.51)	.995	233.42(16.34)	320.50(38.51)	.987
10	342.57(26.03)	291.59(43.10)	.166	312.51(20.54)	311.54(40.23)	.490	206.93(12.06)	352.80(31.66)	1.000
11	320.44(26.89)	306.52(41.77)	.402	261.25(18.64)	341.93(35.75)	.976	236.31(13.56)	338.37(34.14)	.999
12	266.38(23.48)	296.70(41.68)	.730	281.93(25.38)	317.41(40.65)	.765	167.58(10.76)	355.04(30.65)	1.000
13	468.17(31.83)	273.23(42.57)	.000	276.54(17.25)	338.87(37.29)	.936	244.19(14.08)	349.79(31.66)	1.000
14	278.95(25.68)	202.78(41.37)	.071	204.50(20.78)	319.24(40.88)	.994	157.97(9.96)	337.60(34.50)	1.000
15	281.54(22.84)	243.27(44.87)	.230	210.23(17.13)	335.86(38.10)	.999	162.31(9.43)	349.67(31.97)	1.000
1	303.67(19.22)	338.10(34.41)	.800	225.06(12.70)	352.29(32.40)	1.000			
2	382.62(19.12)	354.22(30.96)	.223	342.03(16.85)	353.77(30.79)	.627			
3	188.10(11.61)	338.76(35.35)	1.000	210.68(19.04)	307.91(40.51)	.982			
4	216.11(11.41)	349.31(31.96)	1.000	215.26(11.57)	350.17(32.17)	1.000			
5	461.13(19.02)	352.08(31.26)	.001	479.97(22.10)	343.76(33.70)	.000			
6	198.30(11.02)	353.76(31.09)	1.000	192.05(11.43)	347.35(32.55)	1.000			
7	188.32(10.70)	353.78(30.19)	1.000	184.42(10.92)	354.37(30.74)	1.000			
8	217.20(11.60)	338.42(34.70)	1.000	240.18(17.69)	325.66(37.13)	.981			
9	217.86(14.80)	324.29(36.58)	.998	204.99(15.37)	327.08(36.40)	.999			
10	229.09(15.19)	348.64(31.34)	.999	203.47(11.04)	352.89(31.75)	1.000			
11	288.39(18.49)	325.40(37.50)	.811	248.58(16.60)	334.61(34.96)	.984			
12	161.62(8.82)	353.80(30.60)	1.000	163.28(9.60)	352.00(31.63)	1.000			
13	220.50(11.32)	353.90(31.16)	1.000	221.84(12.12)	353.84(30.39)	1.000			
14	164.63(10.31)	347.36(32.05)	1.000	160.77(10.58)	335.36(35.43)	1.000			
15	167.95(9.78)	353.54(31.21)	1.000	164.47(9.66)	348.08(32.11)	1.000			

Table 8 shows the WAIC and LOOIC values for the fitted models. As can be observed, both indices coincided in selecting the OSDCLM as the model that showed the best balance between fit and parsimony.

**Table 8:** Comparison indices for the fitted models

Fitted model	WAIC			LOO		
	$\hbox {elpd}_{{\mathrm{waic}}}$	${ p}_{{\mathrm{waic}}}$	WAIC	$\hbox {elpd}_{{\mathrm{loo}}}$	${ p}_{{\mathrm{loo}}}$	LOOIC
OSDCLM	$-$3379.2	325.4	6758.5	$-$3381.0	327.2	6762.1
OSCLM	$-$3424.6	300.1	6849.3	$-$3426.2	301.7	6852.4
OSLM	$-$3442.3	286.1	6884.6	$-$3443.9	287.7	6887.7
LLTM	$-$3497.0	285.7	6994.0	$-$3498.5	287.2	6997.0
Rasch	$-$3389.2	287.8	6778.4	$-$3390.8	289.3	6781.5

Table 9 shows the expected a posteriori (EAP) estimates, posterior standard deviations, and posterior probability intervals of the parameters of the OSDCLM. According to the magnitude of the estimates, the second operation defined in the matrix $\mathbf{W}$ was the most difficult operation at the beginning of the test, followed by the fifth, the fourth, the first, and finally the third. It is interesting to note that the EAP estimates obtained by fitting the LLTM led to a different order of difficulty^3^: ${\hat{\alpha }}_1=-1.009$, ${\hat{\alpha }}_2=0.002$, ${\hat{\alpha }}_3=-0.420$, ${\hat{\alpha }}_4=1.810$, and ${\hat{\alpha }}_5=0.355$. These estimates represent the marginal difficulty associated with each cognitive operation; that is, its difficulty confounded with the practice effect.

**Table 9:** Expected a posteriori (EAP) estimates, posterior standard deviations (SD), and posterior probability intervals ($2.5\%-97.5\%$) of the difficulty ($\alpha _m$) and practice parameters ($\delta _m$ and $\gamma _m$) of the operation-specific differential contingent learning model

	$\alpha _m$					$\delta _m$					$\gamma _m$			
	EAP	SD	$2.5\%$	$97.5\%$		EAP	SD	$2.5\%$	$97.5\%$		EAP	SD	$2.5\%$	$97.5\%$
$\alpha _1$	$-$0.564	0.099	$-$0.762	$-$0.370	$\delta _1$	0.680	0.048	0.586	0.776	$\gamma _1$	0.040	0.042	$-$0.043	0.121
$\alpha _2$	3.527	0.780	2.006	5.070	$\delta _2$	0.724	0.201	0.333	1.121	$\gamma _2$	0.676	0.186	0.303	1.049
$\alpha _3$	$-$2.014	0.197	$-$2.405	$-$1.629	$\delta _3$	$-$0.851	0.085	$-$1.018	$-$0.684	$\gamma _3$	$-$0.548	0.073	$-$0.688	$-$0.403
$\alpha _4$	$-$0.443	0.772	$-$1.964	1.065	$\delta _4$	$-$0.222	0.186	$-$0.582	0.134	$\gamma _4$	$-$0.490	0.172	$-$0.829	$-$0.148
$\alpha _5$	1.073	0.139	0.799	1.344	$\delta _5$	0.329	0.129	0.076	0.577	$\gamma _5$	$-$0.695	0.171	$-$1.030	$-$0.364

The positive sign of the estimates of the $\delta _1$, $\delta _2$, $\delta _5$, and $\gamma _2$ parameters, together with the absence of zero in their corresponding posterior probability intervals, indicated the existence of learning associated with correct responses in operations 1, 2, and 5, and learning associated with incorrect responses in operation 2. Note that the second operation was the most difficult operation at the beginning of the test and, therefore, the most prone to require successive approximations for it to be properly performed. The magnitude of the estimates of the parameters suggested a greater learning effect for the second operation, followed by the first, and finally the fifth. The interpretation of these estimates is straightforward. For instance, responding correctly (incorrectly) to an item in which operation 2 was involved provided a decrease of 0.724 (0.676) in the difficulty of this operation.

The negative sign of the estimate of $\delta _3$, together with the absence of zero in its posterior probability interval, indicated an increase in difficulty during the test associated with operation 3 as a function of correct practice, which may be interpreted in terms of progressive fatigue or loss of attention during the test. The negative sign of the estimates of $\gamma _3$, $\gamma _4$, and $\gamma _5$, together with the absence of zero in their corresponding posterior probability intervals, indicated an increase in difficulty during the test associated with operations 3, 4, and 5 as a function of incorrect practice. These results suggested that those individuals who failed in applying these operations at the beginning of the test increased their failure rate in subsequent items, which may be interpreted as loss of interest and/or attention.

The posterior probability interval of the difference between the $\delta _m$ and $\gamma _m$ parameters (Table 10) indicated that this difference was credibly different from zero for operations 1, 3, 4, and 5. These results explain why the OSDCLM fitted the data better than the OSLM, which assumes no difference between $\delta _m$ and $\gamma _m$ for each *m*. Moreover, the fact that the $\gamma _m$ parameter was credibly different from zero for operations 2, 3, 4, and 5 explains why the OSDCLM fitted the data better than the OSCLM, which assumes that $\gamma _m$ equals zero for all *m*.

**Table 10:** Expected a posteriori (EAP) estimates, posterior standard deviations (SD), and posterior probability intervals ($2.5\%-97.5\%$) of the differences ($d_m$) by operation (*m*) between the practice parameters ($\delta _m$ and $\gamma _m$) of the operation-specific differential contingent learning model

$d_m$	EAP	SD	$2.5\%$	$97.5\%$
$d_1$	0.640	0.050	0.543	0.735
$d_2$	0.048	0.069	$-$0.088	0.183
$d_3$	$-$0.303	0.066	$-$0.430	$-$0.179
$d_4$	0.268	0.075	0.124	0.416
$d_5$	1.024	0.189	0.646	1.403

**Fig. 1:**
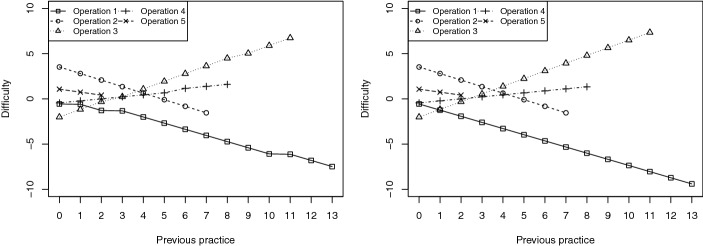
Difficulty of the five cognitive operations as a function of previous practice for subjects $i = 1$ (left) and $i = 2$ (right)

Figure 1 shows the difficulty of the cognitive operations as a function of previous practice for the first two subjects in the response matrix ($i=1$ and $i=2$), whose response patterns were 010101111110111 and 111111111111111, respectively. It should be noted that previous practice equals zero the first time an operation appears in the test. The figure illustrates the decrease in difficulty during the test in operations 1, 2, and 5 for the two subjects. Note that the difficulty throughout the test of operation 2, as well as that of operation 5, was the same for both subjects because their response patterns to the items involving said operation were the same. By contrast, the difficulty of operation 1, as well as that of operations 3 and 4, evolved slightly differently for the two subjects because their response patterns to the items involving said operation were not the same.

### Conclusions

This study illustrates the utility of the proposed model for investigating a variety of practice effects in real data. The best fitting model was the OSDCLM, which suggests the presence of different practice effects in the data derived from correct and incorrect responses. Specifically, learning effects associated with correct responses were observed for operations 1, 2, and 5, whereas a learning effect associated with incorrect responses was observed for operation 2. Additionally, a fatigue effect associated with correct responses was observed for operation 3, whereas fatigue effects associated with incorrect responses were observed for operations 3, 4, and 5.

## Discussion

The purpose of the present work was to introduce a new explanatory item response model for the detection and measurement of differential contingent learning effects during psychometric tests due to the repeated use of the operations involved in the items. To that end, a Bayesian approach was adopted for model estimation and evaluation. The performance of the proposed framework was illustrated with a simulation study and an empirical application. The simulation study demonstrated the accuracy of the MCMC algorithm in parameter recovery as well as the good performance of PPMC and the information criterion indices in model evaluation and selection. The empirical study demonstrated the presence of differential contingent practice effects in real assessment data, which illustrates the utility of incorporating previous practice into item response models for correct and incorrect responses, separately. The proposed framework, therefore, has proved its usefulness when there is a suspicion of practice effects during the test and the goal of the researcher is to adopt an explanatory approach to account for the cognitive processes underlying the item responses. The R and RStan scripts used in this work for model estimation and evaluation are available as supplementary material to this paper.

Nevertheless, it is worth highlighting that the proposed model, as presented in this paper, is based on strong assumptions that might not always be justified. The main assumptions are inherited from the LLTM and the OSLM. Specifically, the LLTM assumes that item difficulty can be linearly decomposed into the difficulties of a well-defined set of operations, and that said difficulties are constant throughout the test and equal for all examinees. In the OSLM, by contrast, the difficulties are allowed to vary linearly as a function of practice, although they are still assumed to be equal for all examinees. These assumptions are highly restrictive and may lead to incorrect results when the assumed operations do not truly reflect the way in which individuals actually solve the items, when the practice effects have a more complex pattern, or when there are individual differences in the practice effects.

The proposed model is more flexible in that it accounts for differential contingent practice effects. In this regard, the model allows for different patterns of change in the difficulty associated with the cognitive operations throughout the test as a function of the persons’ particular response patterns. However, the model still assumes that item difficulty is exclusively determined by the cognitive operations involved in the item, an assumption that may not hold in all cases. For instance, other item properties, such as those related with drawing features in figural items, may also have an influence on item difficulty in certain types of tests. Nevertheless, provided that the researcher is able to operationalize these features, they could be incorporated into the matrix $\mathbf{W}$ to account for their associated effects (e.g., Lozano & Revuelta, 2020). Likewise, learning effects during the test are still assumed to be completely explained by the accumulated practice in the assumed operations, which may be a strong assumption for tests where there are other learning sources to consider (e.g., becoming familiar with test instructions, item response format, item time limit, etc). Additionally, the practice effects are still assumed to be linear throughout the test, which must not necessarily be the case. For instance, a learning effect may show a quadratic trend, with a smaller effect at the beginning of the test and a more pronounced effect toward the end, or vice versa. In such a case, a nonlinear variant of the model, such as that proposed by Spada (1977) and Spada and McGaw (1985) for the OSLM, may be useful.

The model also makes the assumption that practice effects do not differ across items as a function of item difficulty. In this regard, the amount of learning or fatigue derived from performing an operation in a difficult item or in an easy one is assumed to be the same. Although this assumption may be true for many educational and psychological tests in which the items do not show a wide range of difficulty (such as the fraction arithmetic test used in the present study: Range $= .310-.795$, $\hbox {Var} = 0.023$, $\hbox {Sd} = 0.151$), it may not hold for tests with greater variability in item difficulty. In such cases, if there is a suspicion of interaction effects between operations combined in the same items, it may be useful to incorporate the corresponding product terms into the matrix ${\mathbf{W}}$ to account for the extra difficulty and practice effects derived from said interactions instead of using an additive model.

Finally, unlike the OSLM, the model accounts for individual learning patterns based on the persons’ particular response patterns to the items. However, the model still assumes that the learning effects are the same for all examinees, which may be a too restrictive assumption for particular sets of data. In this regard, future studies may be directed to extend the proposed framework to incorporate individual differences in learning (e.g., Embretson, 1991; Rijmen et al., 2002). Regarding future research, it would also be interesting that future studies investigate the influence of practice effects on dimensionality assessment and, more particularly, on over-factoring.

In summary, the proposed model has demonstrated its usefulness in detecting and measuring learning effects during a psychometric test, providing a promising range of applicability. In this regard, the model may be useful in a variety of settings. For instance, the model allows for the assessment of competence acquisition in developmental and educational contexts (e.g., Spada, 1977; Spada & McGaw, 1985), for the substantive analysis of the processes underlying the item responses (e.g., Lozano & Revuelta, 2020, 2021), or for the study of differences in learning ability between populations (e.g., normal vs impaired, children at different developmental stages, etc.). However, the model may also bring novel and interesting methodological applications in the field of adaptive testing. Based on a prior assessment of the difficulty and practice effects associated with each cognitive operation, the model allows for on-the-fly estimation of the difficulty that an item would show in any position within the test as a function of the operations involved in the item and the person’s response pattern to previous items. This opens the door for future studies to investigate the applicability of the model to deal with practice effects in computerized adaptive testing.

## Supplementary Information

Below is the link to the electronic supplementary material.

Supplementary material 1 (rar 4 KB)

## Footnotes

1 In the OSCLM, this vector is defined as ${{\varvec{r}}}_t'={{\varvec{x}}}_t'(\mathbf{W};\mathbf{V}_t)$, whereas in the OSDCLM, ${{\varvec{r}}}_t'={{\varvec{x}}}_t'(\mathbf{W};\mathbf{V}_t;\mathbf{U}_t)$.

2 In the OSCLM, ${{\varvec{\xi }}}=({\varvec{\alpha }},{\varvec{\delta }})$, whereas in the OSDCLM, ${{\varvec{\xi }}}=({\varvec{\alpha }},{\varvec{\delta }},{\varvec{\gamma }})$.

3 The circumflex represents the EAP estimates.
